# Use of antibiotics in the early COVID-19 pandemic in Poland, the Netherlands and Spain, from erraticism to (more) logic

**DOI:** 10.1007/s00228-024-03726-1

**Published:** 2024-07-17

**Authors:** Aleksandra Opalska, Helga Gardarsdottir, Marcel Kwa, Jadwiga Wójkowska-Mach, Monica Sabate, Maria Elena Ballarin, Mark de Groot, Hubert Leufkens

**Affiliations:** 1 Division Pharmacoepidemiology and Clinical Pharmacology, Utrecht Institute for Pharmaceutical Sciences, Faculty of Science, Utrecht University, Utrecht, Netherlands; 2 Department of Clinical Pharmacy, Division Laboratories, Pharmacy and Biomedical Genetics, University Medical Center Utrecht, Utrecht, Netherlands; 3 Department of Pharmacovigilance, Medicines Evaluation Board, Utrecht, Netherlands; 4 Department of Microbiology, Faculty of Medicine, Jagiellonian University Medical College, Crakow, Poland; 5 Department of Clinical Pharmacology, Vall d’Hebron University Hospital, Vall d’Hebron Barcelona Hospital Campus, Barcelona, Spain; 6 Clinical Pharmacology Research Group, Vall d’Hebron Research Institute (VHIR), Vall d’Hebron University Hospital, Barcelona, Spain; 7 UPOD, Central Diagnostic Laboratory, Division Laboratories, Pharmacy and Biomedical Genetics, Utrecht, Netherlands; 8 Directorate-General for Health and Food Safety, European Commission, Brussels, Belgium

**Keywords:** COVID-19, Rational use of antibiotics, Hospitalization

## Abstract

**Introduction:**

In the Spring of 2020, the world was hit with unparalleled impact by the coronavirus pandemic. Antibiotics were widely used, even without good rationale. The aim of our study was to compare the use of antibiotics in patients with confirmed COVID-19 from three hospitals across Europe (Poland, the Netherlands and Spain) between two subsequent periods in the early days of the pandemic.

**Method:**

We analysed data (antibiotics used and variation in the use of antibiotics, patients, admission and disease-related characteristics) from 300 patients admitted in three hospitals (University Hospital in Cracow, University Medical Center in Utrecht and Vall d’Hebron University Hospital in Barcelona) with confirmed infection of SARS-CoV-2 during Q1 2020 and Q4 2020.

**Results:**

There was ample variation in terms of patient mix and outcomes across the 3 hospitals. The majority of patients (225 out of 300) in all 3 hospitals received at least 1 antibiotic during the hospitalisation period. A minority of patients (68 out of 300) had their bacterial test results positive during their hospitalisation period. Throughout the 2 study periods, third-generation cephalosporins (ceftriaxone in 170 out of 300 patients) emerged as the most commonly used class of antibiotics. There was an apparent shift towards more rational utilisation of antibiotics, in all three hospitals.

**Conclusions:**

Our study shows that during the early stage of COVID-19 pandemic in 2020, antibiotics were frequently used in three European teaching hospitals despite the relatively low incidence of microbiologically confirmed bacterial infections. While in the early days of the COVID-19 pandemic antibiotic prescribing was full of trial and error, we could also confirm a learning curve over time.

## Introduction

In the Spring of 2020, Europe and the rest of the world were hit with unparalleled impact by the coronavirus pandemic (COVID-19). The COVID-19 pandemic was linked to the severe acute respiratory syndrome coronavirus 2 (SARS-CoV-2), firstly identified in China in December 2019 [1]. The virus quickly spread through Europe, and according to the European Centre for Disease Prevention and Control (ECDC), there were many millions of cases and significant numbers of COVID-19-related fatalities in Europe. The spread occurred in various time waves in the period 2020–2021 and with variable epidemiology across European countries [2].

In the early days of the COVID-19 pandemic, healthcare professionals were facing unprecedented challenges to help patients affected by COVID-19 with only limited diagnostic and therapeutic options. There were many uncertainties, time was precious and empirical, even without clinical evidence of efficacy and safety, was more the rule than the exception. Also, antibiotics—antimicrobial drugs, but without antiviral activity—were widely used, even without good rationale [3, 4]. Actually, antibiotics were among the most frequently administered medicines in patients with COVID-19. High prevalence of antibiotic use in COVID-19 was widely reported, varying from 70 to 90% [5–8]. Various reasons for starting antibiotic therapy in COVID-19 were stated, i.e. prophylaxis and treatment of bacterial co-infections or secondary infections. Also, experimental therapies, many of these unjustified, with other medicines (e.g. combination of azithromycin with hydroxychloroquine) were reported [9, 10]. According to the Dutch Working Party on Antibiotic Policy, bacterial co-infection upon admission with COVID-19 could only be confirmed in 3.5% of patients, while bacterial secondary infections during hospitalisation occurred in up to 15% of patients [11]. A large UK study that analysed the use of antibiotics in patients hospitalised during the first wave found that the majority of patients received antibiotics despite not having confirmed bacterial infections. The study concluded that bacterial infections were rare and more likely to be secondary infections [12].

To conclude, the beginning of the COVID-19 pandemic in 2020 was full of trial and error, hopes and promises, but short on data and evidence. Doctors and hospitals were taken largely by surprise. Nevertheless, during the course of the COVID-19 pandemic, healthcare systems were also successful in learning and developing more rational antibiotic policies [13–15].

**Fig. 1:**
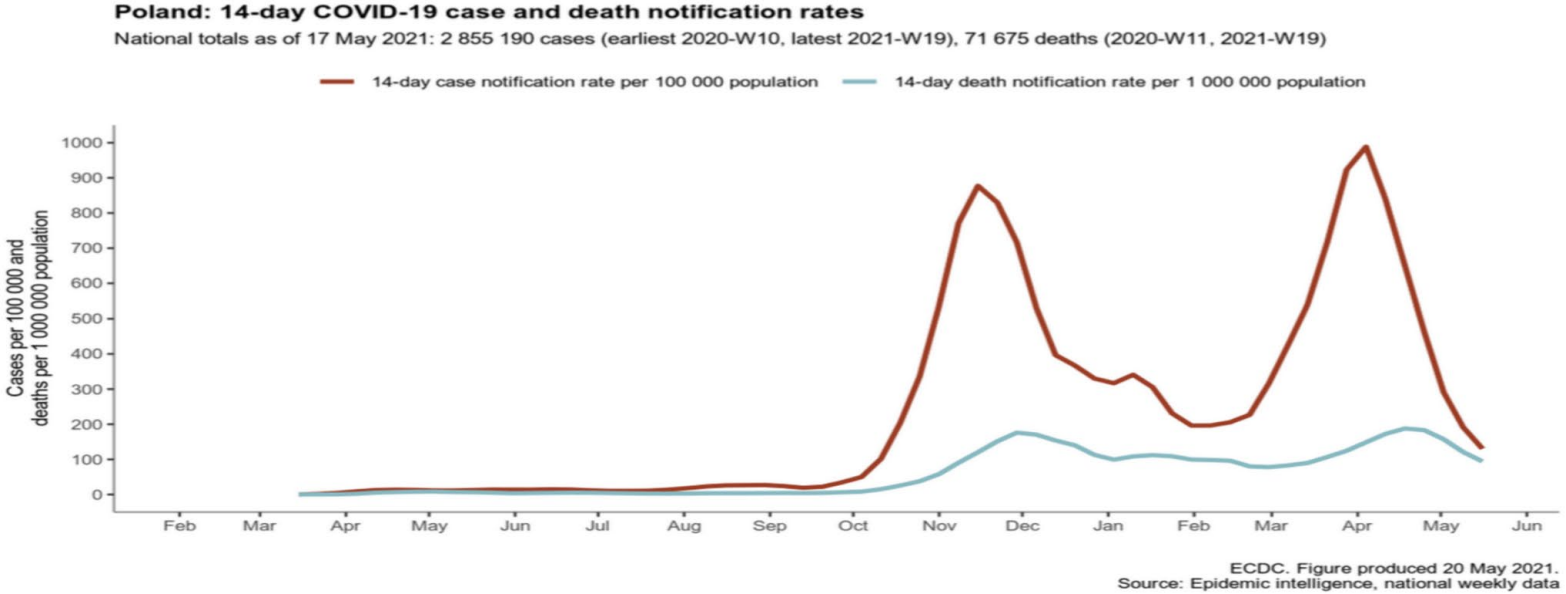
The ECDC overview of COVID-19 cases in Poland

**Fig. 2:**
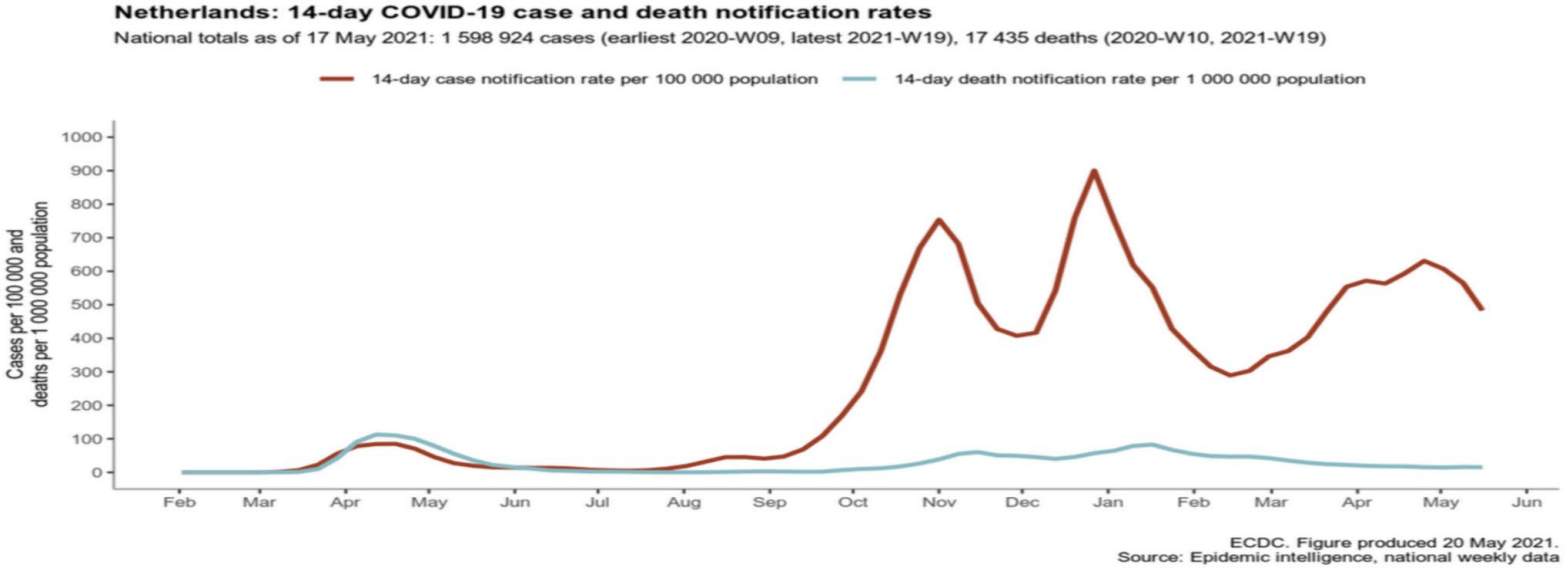
The ECDC overview of COVID-19 cases in the Netherlands

**Fig. 3:**
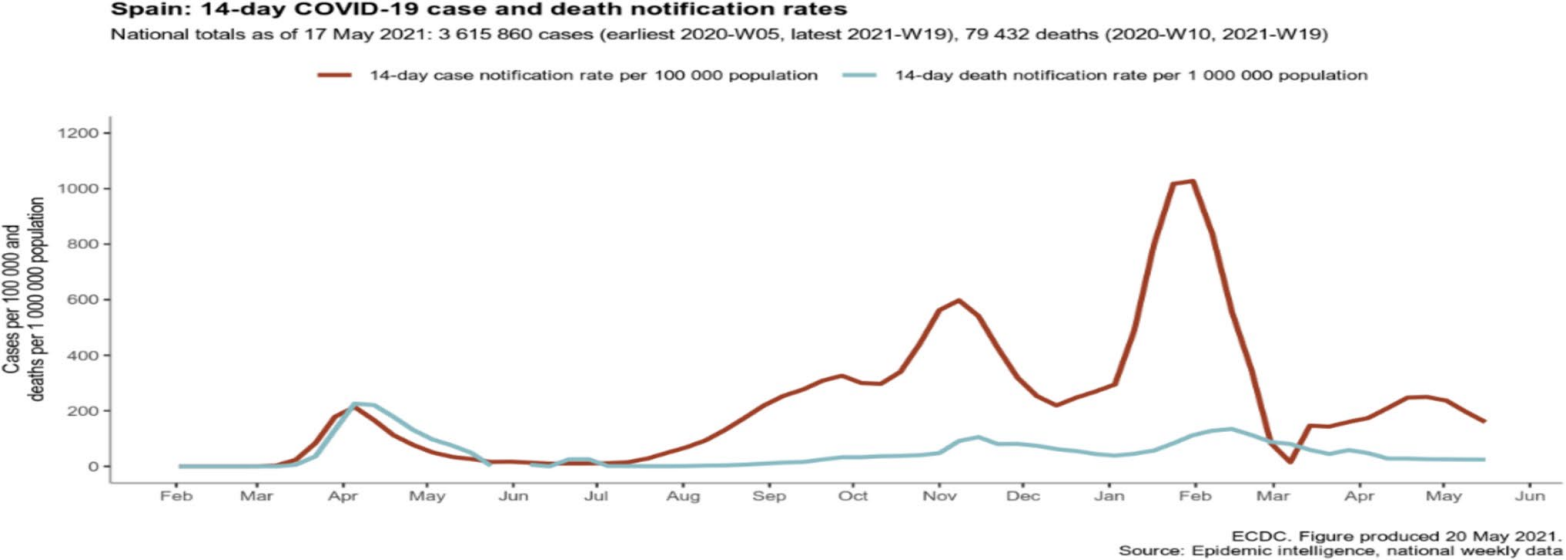
The ECDC overview of COVID-19 cases in Spain

The aim of our study was to compare the use of antibiotics in patients with confirmed COVID-19 from three hospitals across Europe (Poland, the Netherlands and Spain) between two subsequent episodes of the COVID-19 pandemic, i.e. Q1 2020 and Q4 2020. The first confirmed case in Poland was on 4 March [16], in the Netherlands on 27 February 2020 [17] and in Spain on 31 January 2020 [18]. In Spain and in the Netherlands, the first sharp increase of cases was already noticed in the Spring of 2020 (Figs. 1, 2 and 3). This was not seen in Poland.

## Methods

### Setting, study design and participants

This was a retrospective study conducted in three hospitals in Europe: University Hospital in Cracow in Poland (UHC), University Medical Center in Utrecht in the Netherlands (UMCU) and Vall d’Hebron University Hospital in Barcelona in Spain (VHUH). All 3 hospitals have over 1000 beds, considered top-edge academic health hospitals in their respective countries, all three with front-running functions in tackling the disease burden during the COVID-19 pandemic. Data for this study was retrieved from their respective electronic healthcare systems and included the ICD-10 codes; however, at the beginning of the COVID-19 pandemic, the internal codes were used until an ICD-10 code was assigned for COVID-19 infection.

### Study design and participants

In all the 3 hospitals, the first subsequent 50 patients, 18 years old and above, hospitalised with confirmed (positive PCR test result for SARS-CoV-2) infection of SARS-CoV-2 during the first (Q1 2020) and the first subsequent 50 patients during a second period (Q4) were included in the study. Patients with hospital-acquired COVID-19 were eligible for inclusion in this study. Starting date of the analysis in each hospital (the first official/reported patient that was hospitalised including hospital acquired COVID-19 in each hospital) could be different.

In UHC (PL), the first subsequent 50 patients admitted from 13 February 2020 onwards were included. The 50th patient during the first period was admitted on 27 March 2020. During the second wave (t0 + 9 months), the first consecutive 50 patients admitted starting from 12 November were included. The 50th patient during the second period was admitted to the hospital on 14 November 2020. In the UMCU (NL), the first subsequent 50 patients admitted from 27 February 2020 onwards were included in the analysis. The 50th patient during the first period was admitted to the hospital on 16 March 2020. During the second wave (t0 + 9 months), the first consecutive 50 patients admitted starting from 28 November were included. The 50th patient during the second period was admitted to the hospital on 1 December 2020.

In the VHUH (ES), the first subsequent 50 patients admitted from 2 March 2020 onwards were included. The 50th patient during the first period was admitted to the hospital on 14 March 2020. During the second wave (t0 + 9 months), the first consecutive 50 patients admitted starting from 1 December 2020 were included. The 50th patient during the second period was admitted to the hospital on 14 December 2020.

### Exposure to antibiotics

Exposure to antibiotics was ascertained for each included patient. Antibiotic use was defined as treatment with at least one antibiotic course during hospitalisation. For each antibiotic, we ascertained the start and end date of therapy, duration of total antibiotic therapy, the class of antibiotic administered and the number of different antibiotics that were administered during the hospitalisation stay. The duration of antibiotic therapy was assessed in days using start and end date of the specific antibiotic. Antibiotics were divided into the following classes based on the Anatomical Therapeutic Chemical Classification (ATC): polymyxins (ATC J01XB); carbapenems (J01DH); glycopeptides (ATC J01XA); imidazole derivatives (ATC J01XD); lincosamides (ATC J01FF); fluoroquinolones (ATC J01MA); combinations of sulfonamides and trimethoprim, including derivatives (ATC J01EE); beta-lactam antibacterials and penicillins (ATC J01C), including penicillins with extended spectrum (ATC J01CA), beta-lactamase-sensitive penicillins (ATC J01CE) and beta-lactamase-resistant penicillins (ATC J01CF); combinations of penicillins, including beta-lactamase inhibitors (ATC J01CR); other aminoglycosides (ATC J01GB); macrolides (ATC J01FA); monobactams (ATC J01DF); first-generation cephalosporins (ATC J01DB); other cephalosporins and penems (ATC J01DI); second-generation cephalosporins (ATC J01DC); nitrofuran derivatives (ATC J01XE); imidazole derivatives (ATC J01XD); intermediate-acting sulfonamides (ATC J01EC); tetracyclines (ATC J01AA); and other antibacterial medicines (ATC J01XX).

### Other co-variables

Other co-variables extracted for this study included patient, admission and disease-related characteristics. Patient-related characteristics included gender and age at admission. Admission characteristics covered the days of hospitalisation. Disease-related characteristics included death during hospitalisation, if applicable, and information on a positive bacterial test of secondary bacterial infection result that was performed during hospitalisation. No data on the pre-admission period were available for analysis.

### Data collection and research ethics

The analysis was carried out using healthcare data electronically registered in UHC (PL), UMCU (NL), VHUH (ES) and the hospital pharmacies. All data used and analysed for this study was anonymised. The study protocol was approved by the Ethics Committees of UHC (PL) (Nr 1072.6120.2.2021, date of approval 20 January 2021) and VHUH (ES) hospital (ATB-COV-19–2022-01, date of approval 25 April 2022). After review of the study protocol and data management plan, the UMCU (NL) Medical Research and Ethics Committee concluded that this study falls outside the scope of the Medical Research Involving Human Subjects Act and provided a waiver.

## Results

We analysed data from 300 patients admitted in 3 European hospitals in 2 different periods in 2020. There was ample variation in terms of patient mix and outcomes across the three hospitals. The minority of patients in all three hospitals were females, and the youngest patient was 26 years old and the oldest was 96 for both periods. The highest death rate (32%) was observed in VHUH (ES) during the first period of COVID-19.

In UHC (PL), the median patient age during the first period was 52 years (ranging from 26 to 83 years old), while during the second period, it was 61 years (varying between 31 and 82 years old). In the UMCU (NL), the median patient age during the first period was higher, i.e. 66 years (ranging between 26 and 88 years old), and the second period 62 years (spanning from 35 to 85 years old). The median patient age during the first period in the VHUH (ES) was the highest (70 years) (ranging from 28 to 96 years), and during the second period, it was 62 years (varying between 36 and 83 years old). The percentage of female patients hospitalised in both periods was similar across all three hospitals (UHC (PL) 45%, UMCU (NL) 36% and VHUH (ES) 41%). In terms of mortality, 8 patients passed away during the first period, compared to 12 during the autumn period in the UHC (PL). Conversely, in the UMCU (NL) and VHUH (ES) hospitals, the mortality rates decreased from 13 patients to 6 and from 16 to 6, respectively. During the first period at UHC (PL), almost half of the patients (38%, 19/50) were hospitalised for more than 28 days, whereas during the second wave, a majority (48%, 24/50) had hospital stays ranging from 8 to 14 days. In UMCU (NL) and VHUH (ES), the majority of patients during both periods were hospitalised for less than 1 week.

The majority of patients (225 out of 300) in all 3 hospitals received at least 1 antibiotic during the hospitalisation period. A minority of patients (68 out of 300) had their bacterial test results positive during their hospitalisation period (Table 1).

**Table 1:** Characteristics of patients with COVID-19

	UHC (PL)		UMCU (NL)		VHUH (ES)	
	*N* = 100 tot (%)		*N* = 100 tot (%)		*N* = 100 tot (%)	
	1st period *N* = 50	2nd period *N* = 50	1st period *N* = 50	2nd period *N* = 50	1st period *N* = 50	2nd period *N* = 50
Patient characteristics						
- Female	46%	44%	40%	32%	48%	34%
- Age						
18–49 years	42%	18%	16%	18%	8%	16%
50–64 years	32%	40%	24%	44%	30%	28%
≥ 65 years	26%	42%	60%	38%	62%	56%
Admission characteristics						
- Hospitalisation						
≤ 7 days	10%	12%	38%	54%	50%	44%
8–14 days	18%	48%	22%	28%	26%	36%
15–28 days	34%	6%	28%	10%	16%	14%
≥ 29	38%	14%	12%	8%	8%	6%
Disease-related characteristics						
- Bacterial test with positive result	28%	26%	32%	14%	16%	20%
- Death	16%	24%	26%	12%	32%	12%
Antibiotics used						
- Proportion of patients at least one course	62%	86%	86%	70%	94%	52%

**Fig. 4:**
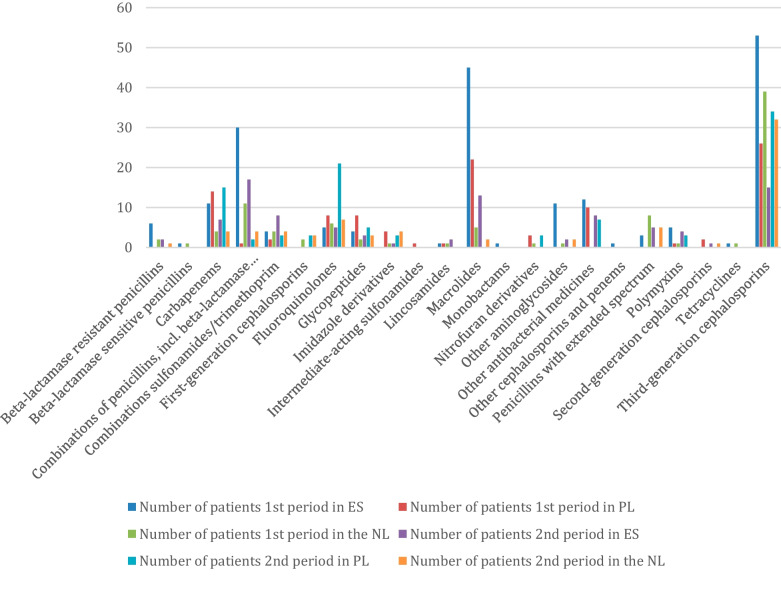
Classes of antibiotics used in hospitalised patients in three hospitals across the two periods

Our analysis revealed notable variations in antibiotic use during the COVID-19 pandemic in the three hospitals. Throughout the two study periods, third-generation cephalosporins, macrolides and combinations of penicillins, including beta-lactamase inhibitors, emerged as the most commonly used classes of antibiotics in all three hospitals (Fig. 4). Among these, ceftriaxone was the prevailing choice, administered to 170 out of all the 300 patients. Azithromycin and meropenem followed, with 75 and 55 patients receiving them, respectively.

In UHC (PL), comparison of the two periods revealed an increase from 62 to 86% of patients receiving antibiotic therapy (Table 1). The most commonly prescribed antibiotic in both periods was ceftriaxone, with 48% (24/50) patients in the first and 64% (32/50) patients in the second period, respectively. Similar proportion of patients received more than 1 antibiotic therapy during hospitalisation during the first wave, 52% (26/31), and the second wave, 56% (24/43).

The study showed that in the UMCU (NL), there was a decrease of antibiotic use from 86% in the first period to 70% in the second. During the first wave in the UCMU (NL), 49% (21/43) patients received more than 1 antibiotic therapy during hospitalisation. In VHUH (ES), a large drop in antibiotic use was seen comparing the two periods, i.e. from 94 to 52%. In the first period, 44 out of 47 patients received more than 1 antibiotic therapy during hospitalisation. This picture changed in VHUH (ES) comparing the two periods from 93 to 77% of patients receiving more than one antibiotic.

Multiple antibiotics were administered to a single patient in all three hospitals. The largest number of antibiotics used in the same patient was observed in VHUH (ES) during the first period (18) and the second period (14). But overall, these were exceptions. In the last column of Table 2, the institutional variation in antibiotic use is reported. In the UMCU (NL) only three antibiotics represented the majority of antibiotic use, stable over the two periods. A sharp drop was seen in UHC (PL), from six to three, while in VHUH (ES), about seven to six different antibiotics were prescribed.

**Table 2:** Variation in use of antibiotics

	No. of patients receiving more than 1 antibiotic		Maximum No. of antibiotics used in one single patient		No. of antibiotics with at least 5 patients (10% of studied cohorts)	
	1st period	2nd period	1st period	2nd period	1st period	2nd period
UHC (PL)	26/31	24/43	7	9	6	3
UMCU (NL)	21/43	14/35	10	9	3	3
VHUH (ES)	44/47	20/26	18	14	7	6

## Discussion

This is an observational study on the use of antibiotics in COVID-19 patients in three different European hospitals in the early days after the onset of the COVID-19 pandemic in 2020. The observed differences are the result of prescribing practices, patient mix and disease burden, epidemiology of the disease and (changes in) hospital admission practices. The observed differences are the result of prescribing practices, patient mix, disease burden, epidemiology of the disease, hospital admission practices, as well as other known and unknown factors. We compared antibiotic use through the lens of different indicators in the very early days of the COVID-19 pandemic and about 9 months later. Our study demonstrates that in all three centres, the second period showed an improved, but modest, and more rational and logical picture. According to the EU guidelines for the prudent use of antimicrobials in humans [19], the rational use of antibiotics constitutes an important component of antimicrobial prudent use and appropriate stewardship strategy.

We saw in UHC (PL) an increase of the proportion of COVID-19 patients with at least one antibiotic course between the two periods of observation. Although this may be partly the result of a change in patient mix (i.e., more elderly patients, higher death rates), it is still an observation that gives concern. In contrast, both in UMCU (NL) and VHUH (ES), we saw a decrease in antibiotic use comparing the two periods, most dramatically in the VHUH (ES). Adherence to national prescribing guidelines, albeit the erratic situation of the pandemic, has been probably more pronounced in UMCU (NL) and VHUH (ES), compared to UHC (PL).

Our study showed that only minority of hospitalised patients in all three hospitals had the positive results of their bacterial tests. Although there may be apparent differences in the percentages of patients with a positive bacterial test (between first and second waves, between centres), this should be interpreted cautiously as they are based on limited numbers. Moreover, without more detailed information about the patients in question (e.g. respiratory status, comorbidity, oxygen level upon admission, ICU admission yes/no, reason for giving antibiotics), there is actually no conclusive explanation for these observations. Obviously, it is possible that at the time of the first wave, when there was still much uncertainty about the causative agent, bacterial origin was also considered with the empirical start of antibiotics for the treatment of possible pneumonia. Or in ICU patients, selective intestinal decontamination can also be started to prevent additional infections. Differences between countries might also be due to differences in national guidelines regarding empirical use of antibiotics when infection is suspected, or in ICU, but there may also have been differences in treatment recommendations for COVID-19 patients at the time depending on the COVID-19 pandemic situation.

The first guideline issued by the World Health Organization issues “The living guideline on the Therapeutics and COVID-19” [20] was published in September 2020. It recommended the use of antibiotics only in specific cases including confirmed bacterial infection. The unprecedented situation during the early stages of the COVID-19 pandemic with many uncertainties regarding effective treatment compelled healthcare professionals to use antibiotics more frequently in treatment of hospitalised patients. According to one research performed in four hospitals in the Netherlands, empiric antibiotic treatment during the early phase of hospitalised patients was frequent [21]. However, the empirical use of antibiotics constitutes a risk factor for the development of antibiotic resistance [22].

We assessed classes of antibiotics used in three European hospitals during two different periods of the initial stage of the COVID-19 pandemic. The most frequently utilised class of antibiotics in all three hospitals was third-generation cephalosporins, with a majority of patients receiving ceftriaxone. This broad-spectrum antibiotic belongs to the WHO “WATCH” group, which are recommended for patients with more severe clinical presentations [23]. Several studies around the world confirm the wide use of ceftriaxone during the COVID-19 pandemic. For example, a Spanish study examining antibiotic use during the COVID-19 pandemic concluded that the usage of ceftriaxone increased by 204% in March 2020 compared to February 2020 [24].

In all three hospitals, the majority of hospitalised patients were female. Some studies have confirmed that COVID-19 appears to affect women less than men [25, 26]. Nevertheless, there were variations in the age distribution of hospitalised patients across the three European hospitals. In the Netherlands and Spain, the majority of patients in both periods were over 65 years old (49 and 59 patients, respectively). In contrast, in the Polish hospital, there were only 34 patients in the same age group in both periods. Additionally, we observed a decrease in the number of patients over 65 years of age hospitalised in the Netherlands and Spain in the second period. The situation in Poland was the opposite, which might be related to the epidemiological picks of the COVID-19 recorded by the ECDC (Figs. 1, 2 and 3). To our knowledge, our study was the first to compare data from three prominent hospitals across distinct European countries—Poland, the Netherlands and Spain. It is important to acknowledge that one of the study’s limitations is the relatively small sample size of patients.

## Conclusion

Our study shows that during the early stage of COVID-19 pandemic in 2020, antibiotics were frequently used in three European teaching hospitals despite the relatively low incidence of microbiologically confirmed bacterial infections. But we also could confirm a learning curve over time. While in the early days of the COVID-19 pandemic antibiotic prescribing was full of trial and error, rational prescribing improved during the course of the crisis. This was seen in all three hospitals, while these experienced very differently the early days of the COVID-19 pandemic. Further analysis is needed in order to understand the additional factors that might have influenced healthcare professionals’ decision to initiate antibiotic therapy. Moreover, the study highlights the importance of establishing and implementing appropriate antibiotic stewardship practices in pandemic situations.

## Data Availability

The data that support the findings of this study are collected in the hospitals according to existing privacy and research integrity rules; they are not openly available, but maybe accessible from the corresponding author upon reasonable request.
