# Correction to: Use of antibiotics in the early COVID‑19 pandemic in Poland, the Netherlands and Spain, from erraticism to (more) logic

**DOI:** 10.1007/s00228-024-03742-1

**Published:** 2024-08-22

**Authors:** Aleksandra Opalska, Helga Gardarsdottir, Marcel Kwa, Jadwiga Wójkowska‑Mach, Monica Sabate, Maria Elena Ballarin, Mark de Groot, Hubert Leufkens

**Affiliations:** 1 Division Pharmacoepidemiology and Clinical Pharmacology, Utrecht Institute for Pharmaceutical Sciences, Faculty of Science, Utrecht University, Utrecht, Netherlands; 2 Department of Clinical Pharmacy, Division Laboratories, Pharmacy and Biomedical Genetics, University Medical Center Utrecht, Utrecht, Netherlands; 3 Department of Pharmacovigilance, Medicines Evaluation Board, Utrecht, Netherlands; 4 Department of Microbiology, Faculty of Medicine, Jagiellonian University Medical College, Crakow, Poland; 5 Department of Clinical Pharmacology, Vall d’Hebron University Hospital, Vall d’Hebron Barcelona Hospital Campus, Barcelona, Spain; 6 Clinical Pharmacology Research Group, Vall d’Hebron Research Institute (VHIR), Vall d’Hebron University Hospital, Barcelona, Spain; 7 UPOD, Central Diagnostic Laboratory, Division Laboratories, Pharmacy and Biomedical Genetics, Utrecht, Netherlands; 8 Directorate‑General for Health and Food Safety, European Commission, Brussels, Belgium

**Correction to: European Journal of Clinical Pharmacology**

10.1007/s00228-024-03726-1

The text should be inserted in the Acknowledgments section:

Jadwiga Wójkowska-Mach was supported by a grant from the the National Centre for Research and Development through the initiative “Support for specialist hospitals in fighting the spread of SARS-CoV-2 infection and in treating COVID-19” (contract number: SZPITALE-JEDNOIMIENNE/18/2020).

The original article has been corrected.

